# Asymmetric Micro‐Evolutionary Responses in a Warming World: Heat‐Driven Adaptation Enhances Metal Tolerance in a Planktonic Rotifer, but Not Vice Versa

**DOI:** 10.1111/gcb.70347

**Published:** 2025-07-17

**Authors:** Shuwen Han, Paul J. Van den Brink, Steven A. J. Declerck

**Affiliations:** ^1^ Department of Aquatic Ecology Netherlands Institute of Ecology (NIOO‐KNAW) Wageningen the Netherlands; ^2^ Aquatic Ecology and Water Quality Management Group Wageningen University Wageningen the Netherlands; ^3^ Laboratory of Aquatic Ecology, Evolution and Conservation, Department of Biology KU Leuven Leuven Belgium

**Keywords:** climate change, copper toxicity, cross‐adaptation, cross‐tolerance, heat stress, micro‐evolutionary adaptation, pollution

## Abstract

The resilience of natural populations in the face of global environmental change is determined by their ability to adapt to multiple, often interacting, stressors. Microevolutionary adaptation to one stressor can either enhance or reduce tolerance to other stressors. In the context of climate change, it is crucial to understand the effect of warming on the tolerance of organisms to additional environmental challenges. Conversely, adaptation to localized stressors, such as pollution, may also affect an organism's capacity to withstand climate change. Here, we investigate how prior adaptation to either high temperature or copper (Cu) contamination influences subsequent tolerance to the other stressor in populations of the freshwater zooplanktonic rotifer *Brachionus calyciflorus* (Pallas, 1766). Using an experimental evolution approach, we subjected populations to either gradually increasing Cu levels, elevated temperature, or control conditions over multiple generations. Subsequently, we conducted a common garden experiment to assess the effect of selection history on population performance. We found that heat‐adapted populations exhibited increased tolerance to Cu, whereas Cu‐adapted populations showed no enhanced tolerance to high temperatures. This form of “asymmetric cross‐adaptation” is likely driven by selection for generalized stress responses associated with heat adaptation, while Cu adaptation selected for more specialized detoxification mechanisms with limited cross‐protection. These findings suggest that the legacy of warming may enhance population tolerance to other stressors, whereas the benefits of adaptation to local pollution may be more constrained. Our study highlights the need to assess the generality of such patterns across taxa and stressor combinations, as this knowledge could inform environmental management strategies in multi‐stressor contexts.

## Graphical abstract

Rapid genetic adaptation is a potentially important mechanism by which natural populations can increase their tolerance to human‐induced environmental stressors. In our study, we demonstrated that zooplankton populations adapted to heat stress exhibited increased tolerance to copper toxicity. However, adaptation to copper did not confer enhanced tolerance to elevated temperatures.

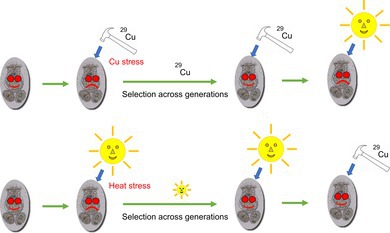

## 1 Introduction

Ecosystems are increasingly impacted by a range of anthropogenically induced stressors that manifest themselves at global (e.g., climate change, ocean acidification), regional, and local scales (e.g., pollution, eutrophication, salinization) (IPCC 2022). Within such systems, the intensity of different stressors tends to fluctuate, causing organisms to experience varying combinations of stressors during the course of time (Ashauer et al. 2017; Gunderson et al. 2016). Given that different stressors can significantly influence each other's effects (Birk et al. 2020; Orr et al. 2020), the consideration of their interactive impacts is crucial for predicting the fate of populations in a multi‐stressor world. For example, in the context of a warming and increasingly polluted world, the need for a better understanding of how increased warming and pollution interact is pressing (Moe et al. 2013). As proposed by Hooper et al. (2013), the physiological mechanisms underlying the interactive effects of toxicants and climatic stressors can be interpreted from two different angles: (1) climate‐induced toxicant sensitivity (CITS), where exposure to a climate‐related stressor makes an organism more sensitive to subsequent toxicant exposure, and (2) toxicant‐induced climate susceptibility (TICS), where toxicant exposure makes an organism more vulnerable to subsequent changes in climatic conditions (Hooper et al. 2013; Huang et al. 2023; Nin and Rodgher 2021; Sokolova and Lannig 2008; Verheyen and Stoks 2019).

Populations in a dynamic multistressor environment may persist through phenotypic plasticity and rapid genetic adaptation. One potentially effective form of phenotypic plasticity in a multi‐stressor environment is cross‐protection, where exposure to an initial “primary” stressor increases an organism's tolerance to a subsequent stressor (Rodgers and Gomez Isaza 2021). In addition, many populations, particularly those with short generation times and large population size, have the capacity for rapid genetic adaptation to changing stressor regimes within ecologically relevant timescales (Kelly 2019; Klerks and Weis 1987). Such microevolutionary adaptations have been observed to significantly alter population responses to single (Declerck et al. 2015; Geerts et al. 2015; Han et al. 2025; Hochmuth et al. 2015) or combinations of stressors (Kelly et al. 2016; Orr et al. 2022). In addition, an increasing number of studies highlight a phenomenon we here term “cross‐adaptation,” in which a population's adaptation to one stressor enhances its tolerance to other stressors, even in the absence of the stressor they have originally adapted to (Janssens et al. 2014; Op de Beeck et al. 2017; Zhang et al. 2018; Zhao et al. 2023), for example, through positive pleiotropic effects. Conversely, adaptation may also result in a reduced ability to cope with other stressors, due to physiological or genetic trade‐offs (“maladaptation”) (Kristiansen et al. 2024; Vereshchagina et al. 2016; Zhou et al. 2025).

The ecological implications of physiological cross‐protection are increasingly recognized (Rodgers and Gomez Isaza 2021). However, the scope and impact of this phenomenon have their limitations, as its initiation is strongly dependent on induction by the primary stressor, and any protective effects are limited in time when exposure to the priming stressor ceases (Rodgers and Gomez Isaza 2023). In contrast, cross‐adaptation is genetically anchored in the populations and does not require induction by the first stressor. Once established, it has the potential to remain effective over many generations (Kristensen et al. 2020). Cross‐adaptation may therefore have a large, hitherto underappreciated contribution to the long‐term resilience of natural populations in a dynamic multi‐stressor environment. As it may also broaden an organism's fundamental niche, it may allow the colonization of new habitats or facilitate range expansions in a rapidly changing world (Debecker et al. 2017). Conversely, maladaptation may reduce population performance and persistence when stressor regimes change (Medina et al. 2007; Moe et al. 2013; Turko et al. 2016).

The long‐term evaluation of the effects of multiple stressor regimes on natural populations is likely to be fully reliable only when the consequences of micro‐evolutionary population responses are considered. Although many studies have explored the combined effects of warming and various stressors (Moe et al. 2013; Noyes et al. 2009; Polazzo et al. 2022), only a few have considered how rapid genetic adaptation to warming may impact an organism's ability to cope with toxic pollutants (Debecker et al. 2017; Dinh Van et al. 2013; Janssens et al. 2014). Addressing this question is, nevertheless, crucial for ecological risk assessments of pollutants in warmer regions and for predicting pollutant impacts under future global warming scenarios (Debecker et al. 2017; Mentzel et al. 2024; Moe et al. 2013). Studies that consider the consequences of microevolutionary adaptation to toxicants on heat tolerance (Ward and Robinson 2005) are even scarcer and, to our knowledge, none consider the consequences of adaptation in both directions. Such studies are, nevertheless, urgently needed, as global warming and its associated heat spells have regional or global consequences that cannot be managed locally, in contrast to many forms of pollution (Rodgers and Gomez Isaza 2023). Understanding the extent to which organisms can rapidly evolve heat tolerance through adaptation to other stressors can inform the management of local stress regimes and help increase the robustness of key populations in the face of global warming.

With this study, we aimed to introduce microevolutionary adaptation into the CITS and TICS discussion. More specifically, we set out to investigate, simultaneously, whether (1) adaptation to heat results in an increased or reduced tolerance to a toxic substance, that is, copper (Cu), and, inversely, (2) adaptation to Cu affects a population's performance in a hot environment. For this, we applied an experimental evolution approach in which we subjected replicate populations of the planktonic rotifer *Brachionus calyciflorus* (Pallas, 1766) to gradually increasing heat or Cu stress and a stressor‐free control environment over the course of many generations. Subsequently, for each of these populations, we studied the effects of these stressors on population growth performance of randomly selected genotypes using a multigenerational common garden experiment with a multifactorial design.

## 2 Materials and Methods

In our experiment, we utilized genotypes (clones) from a natural population of the rotifer *Brachionus calyciflorus s.s*., one of the four species within the *B. calyciflorus* species complex (Michaloudi et al. 2018; Zhang and Declerck 2022). Monogonont rotifers form a functionally important component of freshwater zooplankton and have short generation times, a small body size, and a cyclical parthenogenetic reproduction mode, which alternates between clonal and sexual propagation. These characteristics make them ideal for laboratory evolution experiments and studying genotype‐specific responses to environmental stressors (Declerck and Papakostas 2017; Serra et al. 2019). Clonal lines were established in 2020 by hatching dormant propagules collected from sediments of a freshwater pond (location: 52.02630°, 4.18355°, *The Netherlands*) and subsequently cultivating clonally reproducing populations in the lab. As dormant propagules are sexually produced, each clone is genetically distinct.

### 2.1 Selection Experiment

**FIGURE 1:**
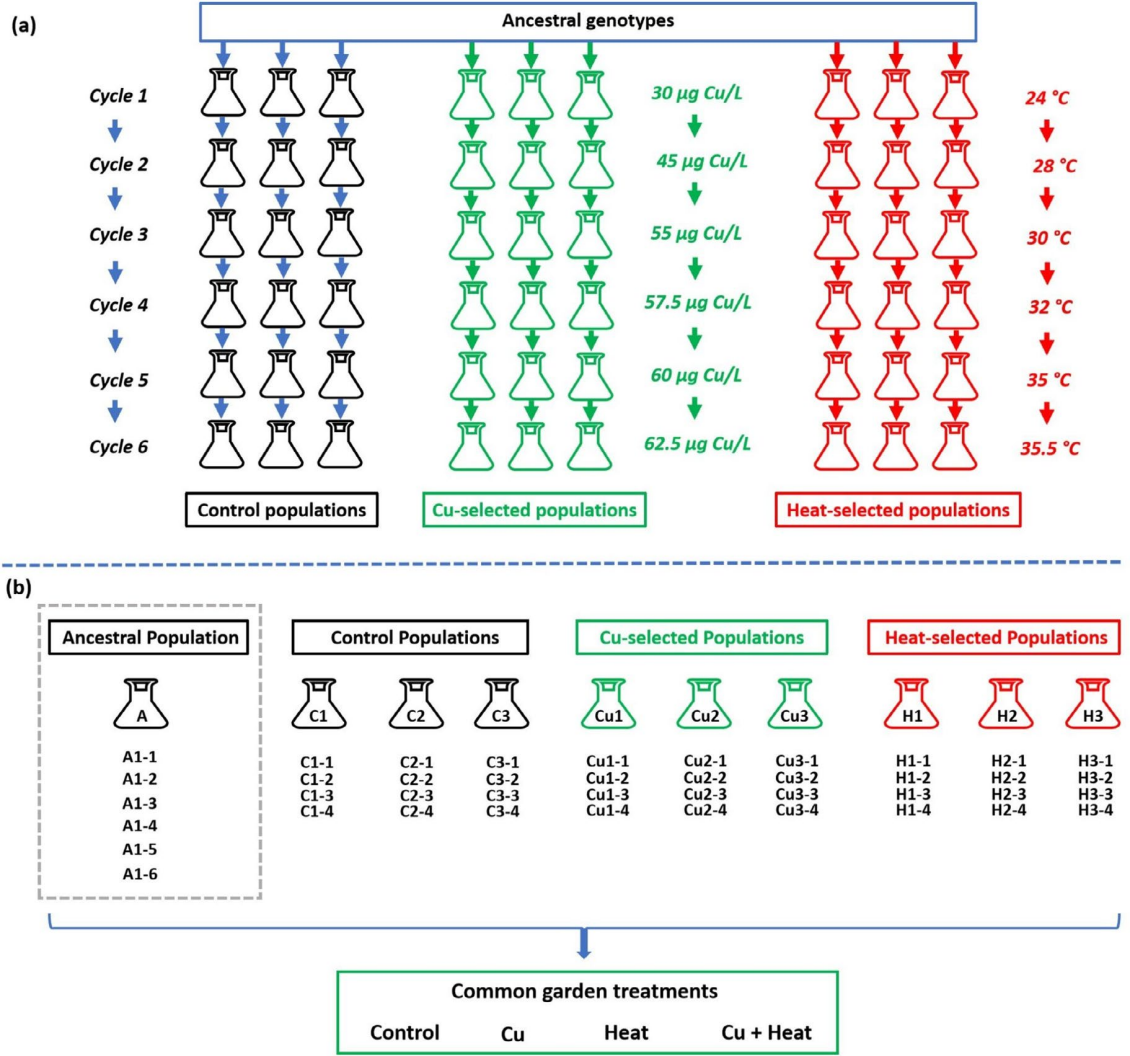
Experimental design. (a) Design of the selection experiment. Using 50 randomly selected genotypes from an ancestral population, we created nine genetically identical populations, three of which subsequently received stepwise increases in Cu concentrations at room temperature (starting at 30 μg·L^−1^ Cu and finishing at 65 μg·L^−1^ Cu at 22°C; “Cu‐selected populations”), three received increasing temperature levels (starting at 24°C and finishing at 35.5°C; “Heat‐selected populations”), whereas the remaining three received no Cu and were kept at room temperature (“Control populations”; 22°C). At each stress level, populations went through a cycle consisting of a period of population growth followed by sexual reproduction (See Figure S1); (b) design of the common garden experiment. For each of the nine populations obtained from the selection experiment, we established four clonal lines from dormant propagules produced after the final cycle in the evolution experiment. In addition, we used six clonal lines from propagules from the ancestral population (“Ancestral”). Experimental populations of all of these lines were individually exposed to control conditions (Control), a high Cu concentration (62.5 μg·L^−1^ Cu; “Cu”), heat (34°C) and the combination of high Cu concentrations and heat (62.5 μg·L^−1^ Cu and 34°C; “Cu+Heat”), resulting in a total of 168 experimental units.

The selection experiment was designed to simulate the microevolutionary responses of natural populations to gradually increasing stress levels. In temperate populations of *B. calyciflorus* , each growing season involves a cycle where a prolonged phase of clonal reproduction is followed by sexual reproduction, leading to the formation of dormant propagules. Upon hatching, these propagules generate new genotypes that re‐establish the population at the start of a new growing season. We initiated the experiment with nine populations, each composed of the same set of 50 clones (Figure 1a). These populations underwent six consecutive cycles during the selection experiment. In each cycle (Figure S1), the population grew clonally until densities were sufficient to induce sexual reproduction (mixis). The duration of each cycle ranged from 7 to 27 days, with higher stress levels leading to slower population growth and a delayed onset of sexual reproduction. At the end of each cycle, dormant propagules were collected and used to establish new, unique genotypes. A new cycle began by re‐initiating the populations with a randomly selected set of 50 clones from the previous cycle. Three replicate populations were maintained under control conditions (“Control populations”; no addition of Cu and heat), three populations were exposed to Cu (“Cu‐selected populations”) whereas the remaining three populations were subjected to increased temperatures (“Heat‐selected populations”) (Figure 1a). Between cycles, both stressors were stepwise augmented (30, 45, 55, 57.5, 60, and 62.5 μg·L^−1^ Cu and 24°C, 28°C, 30°C, 32°C, 35°C, and 35.5°C in cycles 1–6, respectively). Cu was added as CuSO_4_·5H_2_O. Although the Cu concentration applied in our experiment is higher than what is commonly detected in most freshwater ecosystems, it is still within the range reported in polluted systems such as those impacted by mining or industrial discharge (up to 100 μg·L^−1^; e.g., Sanchez et al. 2005). A temperature of 35.5°C may occasionally occur in shallow ponds or lakes during heat waves which are increasingly expected to occur due to global warming, especially at lower latitudes (Woolway et al. 2021).

During the selection experiment, all rotifer populations were grown with the green alga *Chlamydomonas reinhardtii* in WC medium (Guillard and Lorenzen 1972) at a concentration of 1000 μmol·L^−1^ C under constant light. Control and Cu‐selected populations were grown at a temperature of 22°C. Culture flasks (500 mL) were continuously shaken on a rotating plate with 80 rpm. The food suspension in the cultures was daily renewed. Phytoplankton cultures were reared under standardized conditions in continuous cultures (chemostats) at room temperature (22°C).

### 2.2 Common Garden Transplant Experiment

For each of the nine populations in the selection experiment, we haphazardly selected four clonal lines established from dormant propagules produced during the sixth cycle. In addition, to represent the ancestral population, we used six clones from the original set used to initiate the selection experiment (hereafter referred to as the “Ancestral population”). Populations of all these clonal lines were individually exposed to control conditions (“Control” treatment; 22°C and 0.75 μg·L^−1^ Cu), Cu (“Cu” treatment; 22°C and 62.5 μg·L^−1^ Cu), increased temperature (“Heat” treatment; 34°C and 0.75 μg·L^−1^ Cu), and the combination of the latter two (“Cu+Heat” treatment; 62.5 μg·L^−1^ Cu and 34°C) (Figure 1b). The design of the common garden experiment thus consisted of 168 experimental units.

Before starting the common garden experiment, dormant propagules were hatched, and clonal populations were initiated and established under food satiating conditions ( *C. reinhardtii* ; 1000 μmol·L^−1^ C) in the absence of stressors. After an initial phase of upscaling the populations, we created experimental units by transferring 16 randomly selected individuals to wells with 8 mL food suspension ( *C. reinhardtii* ; 1000 μmol·L^−1^ C). Throughout the entire experiment, cultures were maintained by daily re‐initiating populations by transferring 16 random individuals to a fresh food suspension. To purge maternal effects and allow populations to physiologically acclimate to the stress treatments, we gradually increased Cu concentrations and temperature levels over a period ranging between 24 and 30 days until the final experimental target levels were reached. After reaching these concentrations, we monitored the populations for 5 days to ensure that daily population growth rates were positive and constant. Once this was achieved, we continued monitoring the populations during an additional 5 days. Every 24 h, just before transferring 16 individuals to fresh medium, we recorded the number of females, males, dead rotifers, and loose dormant propagules. When counting females, we also made a distinction between females with no eggs, parthenogenetic eggs, non‐fertilized sexual eggs, and dormant propagules. We also counted the number of parthenogenetic eggs per fecund amictic female.

### 2.3 Cu Determination Experiment

To assess the actual Cu concentrations to which rotifer populations had been exposed during the common garden experiment, we performed an additional experiment in which we cultured and monitored rotifer populations exposed to the Control, Cu, and Cu+Heat treatments in exactly the same way as in the common garden experiment. After the daily transfer of rotifers, the remaining medium of each unit (8 mL) was collected, filtered through a glass fiber GF/F filter, and stored at −20°C during a period of 5 days. After the experiment, samples were pooled and the Cu concentrations were analyzed with ICP‐MS by the Soil Chemistry Laboratory of Wageningen University and Research (WUR, Wageningen, the Netherlands). See Supporting Information S1 for more details (Supplementary Method S1).

### 2.4 Data Analysis

Daily population growth was calculated as *r* = (ln*N*_*t*1_−ln*N*_*t*0_)/*t*, where *r* is the exponential population growth rate, *N*_*t*0_ and *N*_*t*1_ are population sizes at the start (*t*0) and end of a 24‐h time interval (*t*1), and t is the length of the time interval (i.e., 1 day). Mortality was calculated as the fraction of females that had died by the end of each time interval. Fecundity was calculated as the mean number of parthenogenetic eggs per egg‐bearing amictic female.

In the Cu+Heat treatment of the common garden experiment, half of the clones of the Control and Cu‐selected populations (i.e., 6 out of 12 clones each) went extinct already during the acclimation phase, in contrast to the clones from the heat‐selected populations, which all persisted until the end of the experiment. These losses resulted in a strong imbalance in the multifactorial design and potentially resulted in a biased representation of population performances of the Control and Cu‐selected populations in the Cu+Heat treatment. To address our key research questions regarding cross‐adaptation, we therefore decided to focus our analysis on a dataset consisting of three common garden treatments only, that is, Control, Cu, and Heat. Although the omission of the Cu+Heat treatment prevented us from testing for Cu × Heat interactions, it preserved maximal statistical power by allowing us to use the full dataset available for these treatments and by securing the balance of our statistical design. Using these data, we applied mixed effects models to test for the interaction of the common garden treatments (Control, Cu, and Heat) with population selection history (Control populations, Heat‐ and Cu‐selected populations). Subsequently, to explore the Treatment × Selection history interaction and test for the existence of cross‐adaptation in both the Cu and Heat treatments, we applied Tukey post hoc analyses controlling for the familywise error rate at *α* = 0.05. Common garden treatments and selection history were specified as fixed factors, whereas “Population identity” (i.e., the identity of populations in the selection experiment from which clones had been extracted) and “Clone” were specified as random factors, with Clone nested within Population identity. Growth rate and fecundity, averaged over the 5 days of the experiment, were analyzed with linear mixed models (LMM), whereas the total of dead versus live individuals and asexual versus sexual females (i.e., females with parthenogenetic eggs vs. females with unfertilized sexual eggs or dormant propagules) counted during the experiment were analyzed with generalized linear mixed models (GLMM), using a binomial distribution and logit link function.

Given that there was only one ancestral population, incorporation of the clones of this population in the previously described analysis would have resulted in a strongly unbalanced design. Yet, to evaluate the effect of the procedure of the evolutionary experiment, we performed a two‐factorial ANOVA testing for an interaction between procedure effect (i.e., Control vs. Ancestral populations) and common garden treatments. In this analysis, clones were used as replicates. Due to the fact that the Ancestral and Control populations were represented by, respectively, 6 and 12 clones, we performed this analysis on a random subset of six Control clones.

As an additional analysis, we also explored the interactive effects of Cu, Heat, and Selection history on population growth rate with a general mixed effects model. To secure a balanced design, we performed this analysis on a reduced dataset by (1) excluding all clones that failed to persist under Cu+Heat conditions and (2) randomly selecting two clones from each of the three original Heat‐selected population replicates. Although this test had reduced statistical power and is biased toward clones surviving the Cu+Heat selection history, it allows us to explore the combined effects of Cu and Heat on the different population types.

All statistical analyses were performed in the R software environment 4.3.3 (R Core Team 2024). Linear and generalized mixed models were performed using, respectively, the “lmer()” and “glmer()” functions in the lme4 package (Bates et al. 2015). To assess the significance of fixed effects and their interaction in the linear mixed models, we used Type III ANOVA with Satterthwaite's approximation for degrees of freedom via the “anova()” function from the “lmerTest” package (Kuznetsova et al. 2017). For generalized linear mixed models, Type III Wald chi‐squared ANOVA was performed using the “Anova()” function from the “car” package (Fox and Weisberg 2019). Tukey post hoc analyses were conducted using the “emmeans” package (Lenth et al. 2025).

## 3 Results

**FIGURE 2:**
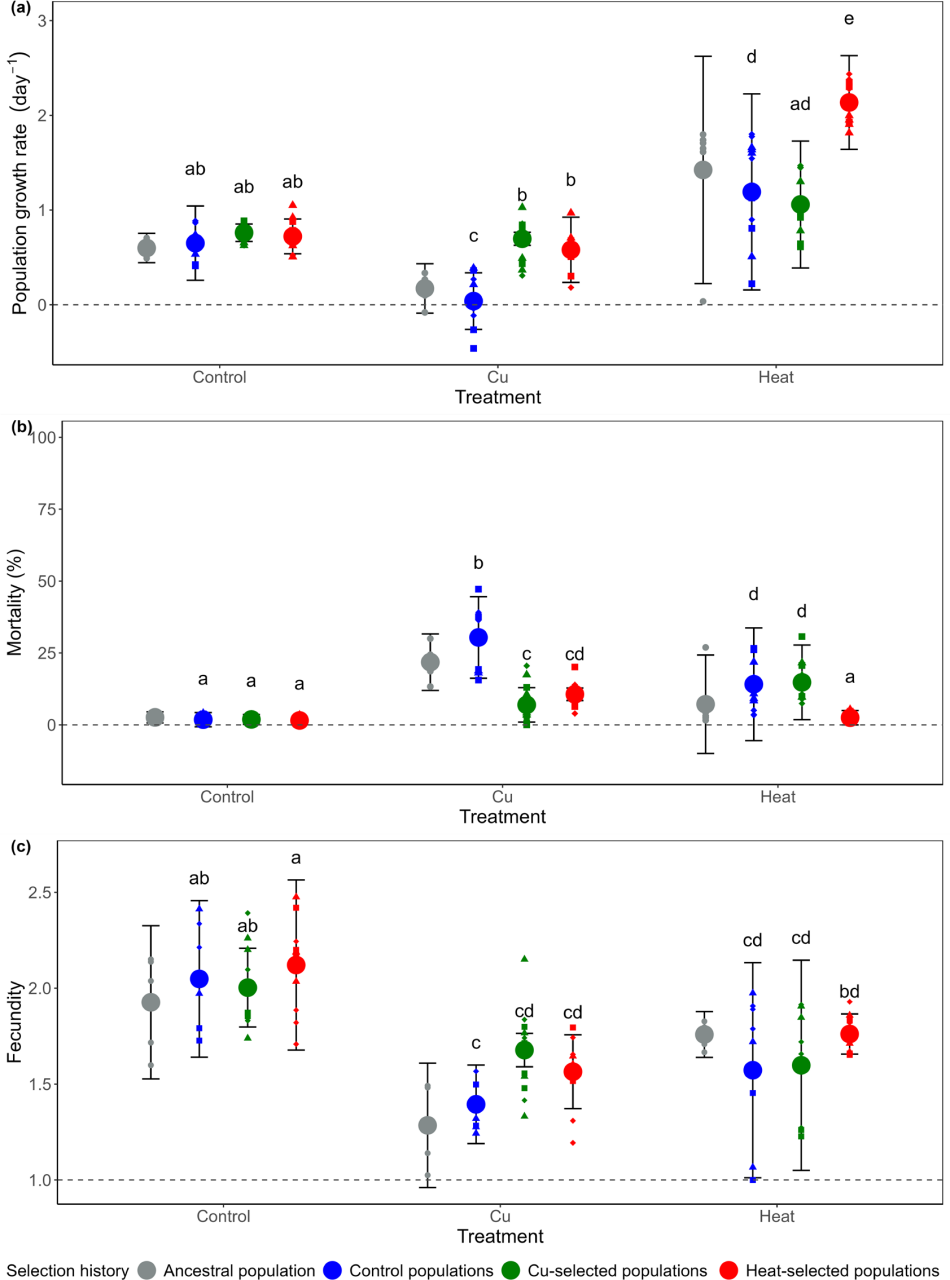
(a) Population growth rate (*r*), (b) mortality, and (c) fecundity of Ancestral, Control, Cu‐, and Heat‐selected populations in response to the Control, Cu, and Heat treatments of the common garden experiment. Symbols and error bars represent means and 95% confidence intervals across population replicates of the selection experiment. Letters denote differences according to post hoc Tuckey pairwise comparisons (alpha = 0.05). Note that, because Ancestral populations did not align with the experimental design, they were not included in this post hoc comparison. Small symbols represent individual clones where different symbol types identify clones that originated from the same population in the selection experiment.

Population growth rate (*r*) was significantly impacted by the interaction between common garden treatments and population selection history (Treatment × Selection history: *F* (4, 64) = 28, *p* < 0.001; Figure 2a; Table 1), indicating that the effect of experimental treatment on population growth rate depended on the selection history of the populations. Compared to the Control conditions, the growth rate of the Control populations decreased by 94% in the Cu treatment (Tukey posthoc comparison: *p* < 0.001; Table S1), whereas no significant reductions were observed for the Cu‐ and Heat‐selected populations. In the Heat treatment, Heat‐selected populations realized a greater population growth than Control and Cu‐selected populations (Tukey test: Heat‐ vs. Control populations, *p* < 0.001; Heat vs. Cu‐selected populations, *p* < 0.001; Table S1), whereas the latter did not differ significantly from each other (Table S1).

**TABLE 1:** General linear mixed effects model for population growth rate and fecundity on the full dataset, after omission if the Cu+Heat treatment.

Dependent variable	Factor	SS	MS	NumDF	DenDF	*F*	*p*
Population growth rate	CG Treatment (CGT)	19.69	9.85	2	64	179	**< 0.001**
	Selection history (SH)	1.30	0.65	2	6	12	**0.010**
	CGT × SH	6.20	1.55	4	64	28	**< 0.001**
Fecundity	CGT	5.19	2.60	2	60	78	**< 0.001**
	SH	0.08	0.04	2	6	1	0.351
	CGT × SH	0.48	0.12	4	60	4	**0.010**

*Note:* Common garden treatment (“CGT”; levels: Cu, Heat and Control) and Selection history (“SH”; levels: Control populations, Heat and Cu selected populations) were specified as fixed factors, whereas Clone and Population ID served as random factors. Bold *p*‐values refer to significant effects (*α* = 0.05).

According to the two‐way ANOVA testing the effects of experimental procedure and common garden treatment on population growth rate, both Control and Ancestral populations exhibited strong main effects in response to the treatments (Figure 2a; *F* (2, 30) = 36, *p* < 0.001), but there were no significant differences among population types or interaction effects between population type and common garden treatment (*p*‐values > 0.95), indicating that the procedure of the selection experiment itself had no major influence on how populations responded to the experimental treatments.

**FIGURE 3:**
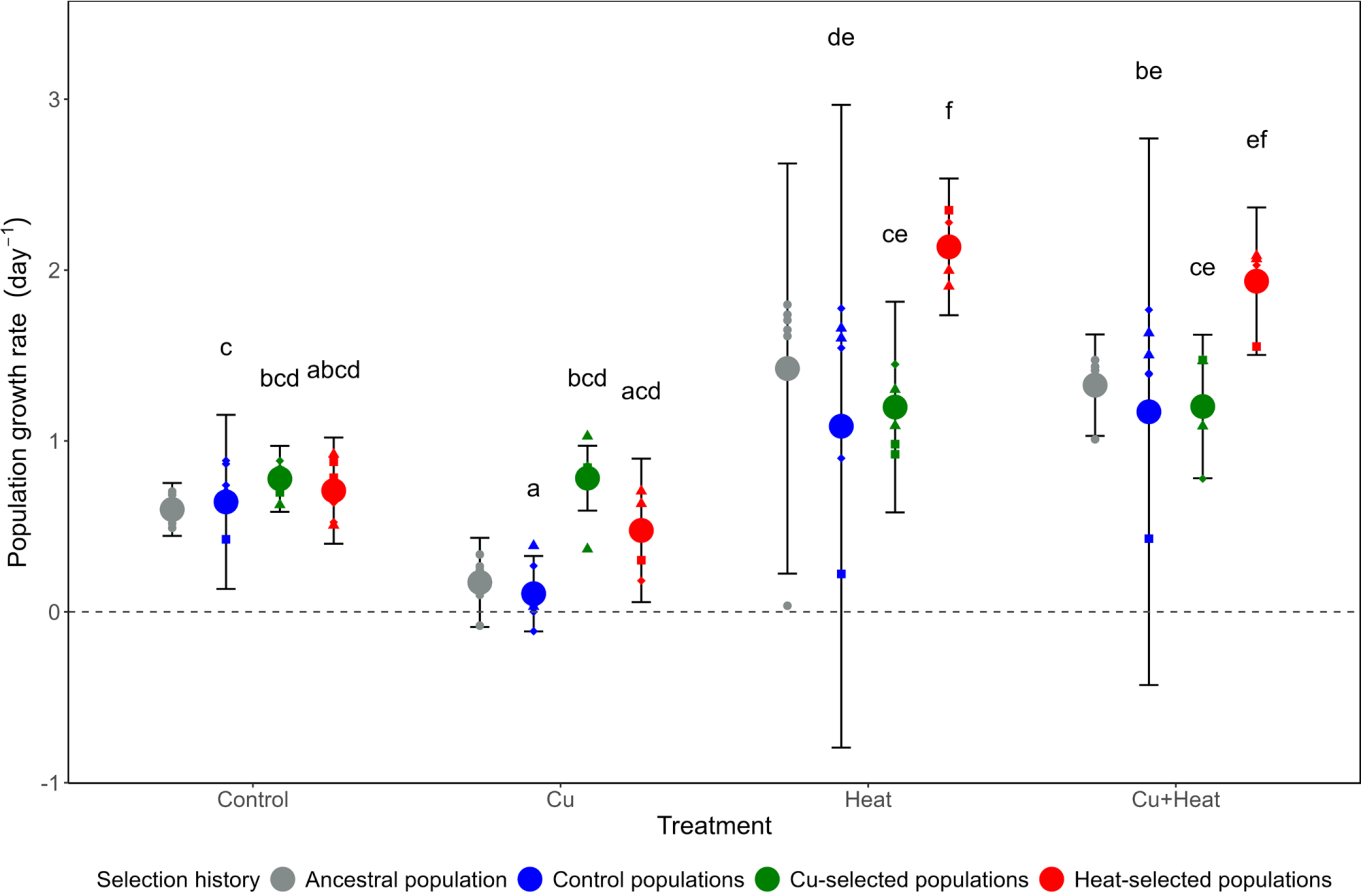
Population growth rate (*r*) of Ancestral, Control, Cu‐ and Heat‐selected populations in response to the Control, Cu, Heat, and Cu+Heat treatments of the common garden experiment, based on the dataset from which we omitted the data of the clones of the Control and Cu‐selected populations that failed to survive the Cu+Heat treatment (see Section 2). Symbols and error bars represent means and 95% confidence intervals across population replicates of the selection experiment. Letters denote differences according to post hoc Tuckey pairwise comparisons (alpha = 0.05).

The LMM analysis based on the reduced dataset (i.e., excluding clones that failed to survive in the Cu+Heat treatment) revealed a significant Cu × Heat × Selection History interaction for population growth rate (*F* (2, 45) = 5, *p* = 0.014; Figure 3; Table S2). Although the Control populations, unlike the Cu‐ and Heat‐selected populations, showed reduced performance due to Cu addition at room temperature—as indicated by lower growth rates in the Cu treatment compared to the Control (Tukey test: Cu vs. Control treatment, *p* < 0.01, Table S3)—this effect was not observed under elevated temperatures. In the Cu+Heat treatment, population growth rates of the Control population were not significantly different from those in the Heat treatment, suggesting that the detrimental impact of Cu was mitigated at higher temperatures. Conversely, whereas Heat‐selected populations showed no response to Cu addition under room temperature conditions, they outperformed both the Control and Cu‐selected populations in the Heat treatment (Tukey test: Heat‐selected vs. Control populations, *p* < 0.01; Heat‐ vs. Cu‐selected populations, *p* < 0.01; Table S3)—but not in the Cu+Heat treatment.

Mortality was found to largely mirror population growth rate (Treatment × Population selection history: *χ* ^2^ = 329, df = 4, *p* < 0.001; Figure 2b, Table 2). In the Control treatment, mortality was very low and unaffected by population selection history. When exposed to the Cu treatment, Control populations showed a higher mortality than populations with Cu and Heat selection histories (Tukey test: Control vs. Cu‐selected populations, *p* < 0.001; Control vs. Heat‐selected populations, *p* < 0.001, Table S4). Compared to the Control treatment, the mortality of the Heat‐selected populations showed no response to the Heat treatment (Tukey test: Control vs. Heat treatment, *p* = 0.2762). Control and Cu‐selected populations responded to the Heat treatment with a significantly higher mortality than in the Control treatment (Tukey test: Heat vs. Control treatments, *p*‐values < 0.001 for both population types). Their mortality in the Heat treatment was also significantly higher than that of the Heat‐selected population (Tukey test: Control vs. Heat‐selected populations, *p* < 0.001; Cu‐ vs. Heat‐selected populations, *p* < 0.001).

**TABLE 2:** Generalized linear mixed effects models (binomial distribution with log‐link function) for mortality (counts of dead vs. alive rotifers) and sexual investment (counts of females with sexual eggs vs. parthenogenetic eggs).

Dependent variable	Factor	*χ* ^2^	df	*p*
Mortality	Intercept	335	1	**< 0.001**
	CG treatment (CGT)	390	2	**< 0.001**
	Selection history (SH)	0	2	0.881
	CGT × SH	329	4	**< 0.001**
Frequency of females with sexual eggs	Intercept	2	1	0.199
	CG treatment (CGT)	211	2	**< 0.001**
	Selection history (SH)	2	2	0.360
	CGT × SH	26	4	**< 0.001**

*Note:* Common garden treatment (“CGT”; levels: Cu, Heat and Control) and Selection history (“SH”; levels: Control populations, Heat‐ and Cu‐selected populations) were specified as fixed factors, whereas Clone and Population ID served as random factors. *χ* ^2^: Type III Wald chi‐square; df: Degrees freedom. Bold *p*‐values refer to significant effects (*α* = 0.05).

Fecundity was significantly impacted by a Treatment × Selection history interaction (*F* (4, 60) = 4, *p* = 0.010; Figure 2c, Table 1). Post hoc comparisons indicated that fecundity was consistently lower under the Cu and Heat treatments compared to the Control treatments across all population types but indicated no differences among population types within treatments (Figure 2c; Table S5). Yet, fecundity of Heat‐selected populations in the Heat treatment was significantly larger than that of the Control populations in the Cu treatment (Tukey test: *p*‐value = 0.012; Table S5).

The GLMM indicated a significant Treatment × Selection history interaction for the fraction of females with sexual eggs (*χ* ^2^ = 26, df = 4, *p* < 0.001; Figure S2, Table 2), but Tukey post hoc comparisons revealed no significant pairwise differences among populations within treatments (Table S6). Compared to the Control treatments, the fraction of females with sexual eggs was significantly reduced in the Heat and Cu treatments (main treatment effect: *χ* ^2^ = 211, df = 2, *p* < 0.001; Figure S2, Table 2).

Actual Cu concentrations in rotifer cultures approached well the concentrations targeted for the different Cu addition treatments in the common garden experiment. Control, Cu, and Cu+Heat treatments equaled 0.75, 54.20, and 56.63 μg·L^−1^ Cu, respectively (Figure S3).

## 4 Discussion

Unique about the design of our study is that it allowed us to study the patterns of cross‐adaptation among two stressors in both directions simultaneously. When exposed to Cu, populations adapted to heat exhibited a fitness advantage compared to Control populations and performed equally well as Cu‐selected populations. In contrast, when exposed to heat, populations adapted to Cu exhibited similar performance to Control populations, indicating that adaptation to Cu neither confers cross‐adaptation to heat nor results in a performance trade‐off (Figure 2a). From a micro‐evolutionary perspective, this pattern of “asymmetric cross‐adaptation” does not seem to provide support for the idea that climate change increases sensitivity to toxins (CITS) or that toxins increase susceptibility to climate change (TICS) (Hooper et al. 2013).

The observed pattern of “asymmetric cross‐adaptation” suggests that cross‐adaptation may depend on the degree of generality of the stress responses that are selected for during microevolutionary adaptation to stress regimes. Heat stress is known to induce cross‐tolerance of organisms to a broad range of other stressors (Rodgers and Gomez Isaza 2023), including metals (Wang et al. 2020), pesticides (Alzahrani and Ebert 2018), or osmotic stress (DuBeau et al. 1998), likely due to the activation of a generalized stress response (Kültz 2005), that involves the upregulation of universally conserved stress proteins, including heat shock proteins, antioxidant enzymes, and DNA repair mechanisms (Sottile and Nadin 2018). Our results suggest that selection by heat stress has promoted genotypes with enhanced generalized stress responses that render them more tolerant not only to heat but also to other stressors like Cu. In contrast, selection by Cu stress appears to have selected for genotypes that are effective in reducing metal stress through more specialized mechanisms that render no advantage against heat stress. Organisms typically deal with metal stress by reducing the active uptake rate of metal ions and through the upregulation of specific detoxification mechanisms (Sokolova and Lannig 2008), such as the expression of metallothionein (Amiard et al. 2006). Although metals are also known to induce generalized stress responses such as ROS scavenging (Ruttkay‐Nedecky et al. 2013) and protein chaperone functions, the lack of impact of Cu adaptation on heat tolerance indicates that these functions have not been induced or effective in the populations exposed to heat.

Studies investigating how organisms cope with the combined stresses of toxins and heat primarily focus on physiological and toxicological mechanisms (Hooper et al. 2013; Mangold‐Döring et al. 2022; Nin and Rodgher 2021; Noyes and Lema 2015; Sanpradit et al. 2024). Our study underscores the critical need to integrate micro‐evolutionary adaptation into this research field (Dinh Van et al. 2013; Op de Beeck et al. 2017; Zhang et al. 2019; Orr et al. 2022). The finding that Cu exposure led to a marked population growth rate reduction in naïve populations, while heat‐selected populations showed no effect from Cu, is striking and suggests that the legacy of warming can have an unexpectedly large impact on the performance of populations when confronted with a new stressor like Cu. While heat stress may pose significant risks and negative impacts on populations, our results suggest that populations that eventually adapt could benefit from increased tolerance to other stressors. Such adaptation may not only increase the persistence of populations in the face of new stress regimes but also expand their fundamental ecological niche, enabling them to thrive in environments with stressor regimes that were previously unsuitable.

These findings point to several questions that warrant further investigation. For how long would such cross‐adaptation persist in the absence of heat stress, and to what extent can it then be sustained by other environmental stressors? And how broadly applicable is this response across taxa and types of stressors? Comparisons of damselfly populations (*Ischnura elegans*) from varying latitudes suggest that populations from warmer climates may exhibit higher tolerance to stressors, such as metals and pesticides, compared to those from colder regions (Dinh Van et al. 2013; Janssens et al. 2014; Op de Beeck et al. 2017). Using a resurrection approach, Zhang et al. (2018) demonstrated that genotypes with a recent history of exposure to heat waves had higher tolerance to Zn at high temperatures than genotypes from ancestral populations. However, contrary patterns have also been reported (Debecker et al. 2017; Dong et al. 2024; Kristiansen et al. 2024; Sumon et al. 2018). In an experimental evolution study with *Daphnia magna* , for example, Zhang et al. (2019) found no impact of heat adaptation on tolerance to zinc nanoparticles. Understanding how adaptation to simultaneously occurring stressors influences patterns of cross‐adaptation is also crucial. Specifically, to what extent are the observed patterns of cross‐adaptation in response to heat and metal stress robust when both stressors act concurrently as selective pressures? Orr et al. (2022) examined the adaptive responses of rotifer populations ( *Brachionus calyciflorus* ) exposed simultaneously to multiple stressors—Cu, temperature, and salinity—and found that such adaptation led to increased synergistic interactions among stressors, likely due to trade‐offs. However, their study used reduced temperature as the thermal stressor, rather than elevated temperature. Conducting similar experiments with heat stress (i.e., increased temperature) would be essential to assess the extent to which trade‐offs constrain cross‐adaptation in multistressor environments under different scenarios of climate warming.

Stressors such as pollutants can be managed locally, unlike large‐scale climate‐related stressors such as warming and heat waves (Rodgers and Gomez Isaza 2023). The concept that genetic adaptation to controllable, localized stressors might enhance tolerance to uncontrollable, large‐scale stressors offers an appealing framework for optimizing stressor management under multiple stress scenarios. However, given that we found no evidence of cross‐adaptation from Cu adaptation to heat stress, our results suggest limited scope for leveraging Cu management to improve population resilience to heat. This aligns with earlier findings reporting neutral or limited cross‐protection effects (Ward and Robinson 2005). While the potential for Cu adaptation to confer tolerance to other stressors (e.g., other metals) remains an open and relevant question, our findings highlight the complexity of predicting cross‐adaptive outcomes and underscore the need for case‐specific evidence when prioritizing stressor management strategies.

The observation that populations with a heat exposure history evolved greater tolerance to Cu toxicity may seem surprising, given that population growth rates were consistently higher in the Heat than Control treatments. The idea that higher growth rates coincide with higher selection pressure due to heat stress at first appears counterintuitive. However, as a major driver of metabolic rate, temperature is quite a unique environmental factor. Warming typically results in an increase of population growth rates because it enhances metabolic rates, resulting in higher somatic growth rates, a faster development, and earlier reproduction (Gillooly et al. 2001). However, concurrent with such increased population growth rates, increased temperatures are likely to also lead to enhanced physiological stress, such as oxidative stress and protein denaturation (Desalvo et al. 2008; Lushchak 2011; Sokolova and Lannig 2008). Therefore, enhanced population growth rates at high temperatures do not necessarily preclude genetic selection for stress‐coping abilities. Several observations indeed indicate that the high‐temperature treatment of our experiment induced substantial heat stress, driving microevolutionary adaptation. First, from our experience and previous studies (Kiemel et al. 2022; Paraskevopoulou et al. 2020), we know that 35.5°C considerably exceeds the optimal temperature range for the studied species (i.e., approximately 25°C). Second, although mortality rates were higher in the Heat than in the Control treatments for Control and Cu‐selected populations, this was not observed for populations with a history of heat selection, suggesting that Heat‐selected populations had evolved stress‐coping mechanisms that reduced sensitivity to heat stress, despite the overall positive effect of heat on population growth rates (Figure 2b). Together with the finding that populations adapted to the Heat treatment also displayed a decreased sensitivity to Cu stress, these observations strongly suggest that the Heat treatment selected for genotypes combining rapid growth under heat stress with an enhanced stress tolerance.

The Cu+Heat treatment enables an assessment of the combined impact of both stressors on populations with diverse selection histories. Clones of all population types persisted throughout the experiment in the Control, Cu, and Heat treatment. In contrast, in the Cu+Heat treatment, half of the clones of the Ancestral, Control, and Cu‐selected populations perished already during the acclimation phase prior to the common garden experiment, indicating that the combination of Cu and Heat created a more challenging environment than any of the other treatments. Yet, none of the Heat‐selected clones were lost in the Cu+Heat treatment of the common garden experiment, an observation that provides additional support for the idea that adaptation to heat increases tolerance to Cu. Surprisingly, however, for the remaining clones of the Ancestral and Control populations, the combination of Heat and Cu did not diminish population growth compared to heat stress alone, indicating that even within populations with identical selection history, clones may differ strongly in the way they cope with stressor regimes (Delnat et al. 2022). For these remaining clones, the relative advantage of Cu adaptation in Cu treatments observed at room temperature disappeared under heat stress. This is unlikely an effect of temperature on the Cu treatment itself because Cu measurements revealed no reduction in Cu concentrations in the Cu+Heat compared to Cu treatment, and the relative toxicity of Cu is expected to show only ignorable variation within the relatively narrow temperature range of our experimental treatments (22°C–32°C) (Sokolova and Lannig 2008; Stumm and Morgan 2013). Instead, the observed lack of negative Cu effects in the Cu+Heat treatment on the population growth of the remaining Ancestral and Control clones suggests physiology‐based heat‐induced cross‐tolerance to Cu. Intriguingly, this parallels the improved tolerance of heat‐adapted populations to Cu observed in Cu treatments at room temperature, suggesting that the latter pattern of cross‐adaptation may have arisen through selection on generalized stress responses akin to those protecting heat‐induced rotifers from Cu stress. Validating this hypothesis, however, would require additional approaches such as transcriptomic analysis, metabolomic profiling, and functional genomics to comprehensively assess changes in gene expression, gene function, and metabolic pathways across treatments and populations (Stanford et al. 2020; Yampolsky et al. 2014).

## 5 Conclusion

Our findings highlight the critical need to integrate microevolutionary perspectives into studies of organisms facing multi‐stressor environments, particularly in the context of climate change, where selection due to warming may have lasting effects on a population's tolerance to future stressors, especially in organisms with short generation times. More broadly, our results demonstrate that adaptation to an initial stressor can confer increased tolerance to subsequent stressors, though this phenomenon is not necessarily reciprocal. Such asymmetry can significantly influence the ecological impact of stressor regimes, making outcomes highly dependent on the sequence in which stressors occur. Our study serves as a proof of concept that, we hope, will inspire future research on cross‐adaptation across multiple species and stressor combinations. Identifying the general patterns of cross‐adaptation could greatly enhance our ability to understand and predict the long‐term effects of stressor regimes on population dynamics, biodiversity, and ecosystem resilience and be instrumental in optimizing strategies for managing environmental stressors.

## Author Contributions

**Shuwen Han:** conceptualization, data curation, formal analysis, funding acquisition, investigation, writing – original draft. **Paul J. Van den Brink:** conceptualization, supervision, writing – review and editing. **Steven A. J. Declerck:** conceptualization, data curation, formal analysis, methodology, project administration, resources, supervision, writing – original draft, writing – review and editing.

## Conflicts of Interest

The authors declare no conflicts of interest.

## Supporting information


Data S1.

## Data Availability

The data that support the findings of this study are openly available in DRYAD at https://doi.org/10.5061/dryad.np5hqc054.
