# Supplementary material for: Asymmetric Micro‐Evolutionary Responses in a Warming World: Heat‐Driven Adaptation Enhances Metal Tolerance in a Planktonic Rotifer, but Not Vice Versa
Source: Glob Chang Biol. 2025 Jul 17;31(7):e70347. doi: 10.1111/gcb.70347 (PMC12268377; doi:10.1111/gcb.70347)
Supplement: Supplementary file 1 — Data S1. [file GCB-31-e70347-s001.docx]

**Supplementary Information: Asymmetric micro-evolutionary responses in a warming world: heat-driven adaptation enhances metal tolerance in a planktonic rotifer, but not vice versa.**

Shuwen Han^1^, Paul J. Van den Brink^2^ and Steven A.J. Declerck^1,3^

1 Department of Aquatic Ecology, Netherlands Institute of Ecology (NIOO-KNAW), P.O. Box 50, 6700AB Wageningen, the Netherlands

2 Department of Aquatic Ecology and Water Quality Management, Wageningen University, P.O. Box 47, 6700 AA Wageningen, The Netherlands

3 Department of Biology, Laboratory of Aquatic Ecology, Evolution and Conservation, KULeuven, Ch. de Beriotstraat 32, 3000 Leuven, Belgium


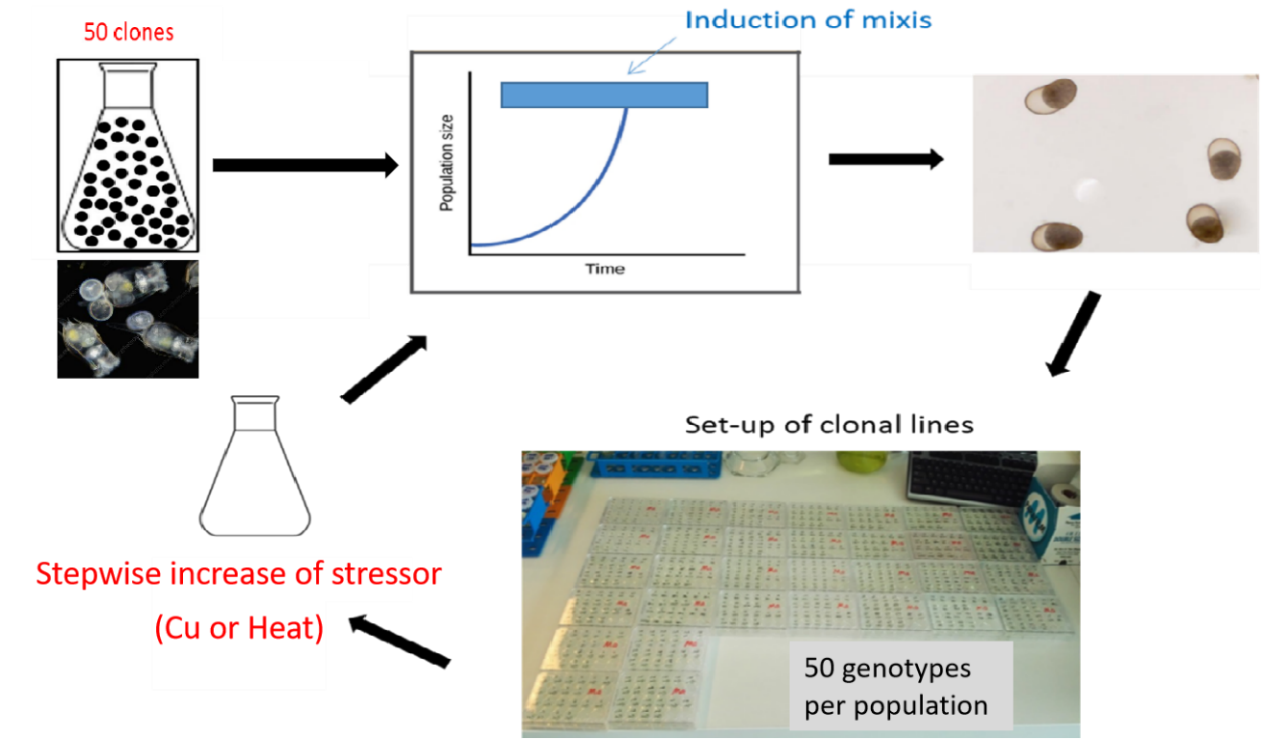


**Figure S1.** Schematic representation of a cycle at a given stress level. Each cycle in the selection experiment was started with a set of 50 clones from the ancestral population (1^st^ cycle) or produced during the previous cycle. First, parthenogenetic populations were allowed to grow exponentially until high population density and food limitation induced sexual reproduction. At the end of each cycle, sexually produced dormant propagules were isolated and hatched to obtain 50 clonal lines per population whereas the remainder were stored for later use. At the start of a new cycle original populations were re-initiated by combining the clonal lines of the previous cycle into a new population and the stress level (Cu or Heat) was increased. Modified from (Zhao et al., 2023).

**Supplementary Method: Cu determination experiment**

From each of the three replicate populations from the Cu-addition treatment in the selection experiment, we established three clone lines from dormant propagules produced during the sixth cycle (62.5 µg·L^-1^ Cu). Populations consisting of a mixture of these clones were exposed to three treatments equal to those applied in the common garden experiment: Control conditions, Cu (62.5 µg·L^-1^ Cu; 22°C) and Cu+Heat (62.5 µg·L^-1^ Cu; 34°C), with three replicates per treatment.

Before starting the experiment, dormant propagules were hatched and clonal populations were initiated and established under food satiating conditions (*C. reinhardtii*; 1000 µmol·L^-1^ C) in the absence of Cu and heat stress. After an initial phase of upscaling the populations, we created experimental units by transferring 18 randomly selected individuals to wells with 9 mL food suspension (*C. reinhardtii*; 1000 µmol·L^-1^ C). Cultures were maintained by daily transferring 18 random individuals to a fresh food suspension. Before the actual experiment, to acclimate populations, we gradually increased Cu and heat levels in the corresponding treatments until the final experimental target concentrations were reached. Subsequently, we monitored populations for a period of 5 days to ensure that daily population growth had stabilized. Every 24 hours, 18 individuals were randomly selected and transferred to fresh medium. Once all treatments had reached the target Cu concentration and heat levels, the collection phase of the experiment began.

After having transferred the individuals, the remaining culture solution was processed to separate the medium from the algae. Firstly, approximately 8 mL of the remaining culture solution was retained and dispensed into four 2 mL centrifuge tubes. Subsequently, the culture medium was centrifuged for 30 minutes by using a high-speed centrifuge with a speed of 14,800 rpm. After the centrifugation process was completed, the supernatant from all four tubes would be gently transferred and pooled into a 50 mL centrifuge tube for storage. For each experimental unit, this was repeated during a period of 5 days and the serially collected medium was pooled in one single centrifuge tube (volume: 25mL) in the freezer at -20 °C. Once the collection was completed, the medium was filtered through a GF/F membrane filter (diameter: 47 mm; pore size; 0.45 µm), using a vacuum pump. The purpose of this step was to ensure complete removal of algae and other particles. The filtered medium was stored at -20 °C until ICP-MS analysis.

**Table S1.** Tukey post hoc pairwise comparisons for population growth rate among Treatment × Selection history level combinations. CGT-SHC refers to specific common garden treatment – selection history combinations.

| **Multiple Comparisons**  **(CGT-SH vs CGT-SH)** | **Estimate** | **Std. Error** | **z value** | **Pr(>\|z\|)** |
| --- | --- | --- | --- | --- |
| ControlCu vs. ControlControl | 0.11291 | 0.13323 | 0.847 | 0.99456 |
| ControlHeat vs. ControlControl | 0.07440 | 0.13323 | 0.558 | 0.99973 |
| CuControl vs. ControlControl | -0.60896 | 0.09828 | -6.196 | **< 0.001** |
| CuCu vs. ControlControl | 0.04927 | 0.13323 | 0.370 | 0.99999 |
| CuHeat vs. ControlControl | -0.06733 | 0.13323 | -0.505 | 0.99987 |
| HeatControl vs. ControlControl | 0.56964 | 0.09996 | 5.699 | **< 0.001** |
| HeatCu vs. ControlControl | 0.40812 | 0.13504 | 3.022 | 0.05772 |
| HeatHeat vs. ControlControl | 1.48831 | 0.13323 | 11.171 | **< 0.001** |
| ControlHeat vs. ControlCu | -0.03851 | 0.13134 | -0.293 | 1.00000 |
| CuControl vs. ControlCu | -0.72187 | 0.13134 | -5.496 | **< 0.001** |
| CuCu vs. ControlCu | -0.06364 | 0.09570 | -0.665 | 0.99902 |
| CuHeat vs. ControlCu | -0.18024 | 0.13134 | -1.372 | 0.89739 |
| HeatControl vs. ControlCu | 0.45673 | 0.13323 | 3.428 | **0.01592** |
| HeatCu vs. ControlCu | 0.29520 | 0.09821 | 3.006 | 0.06041 |
| HeatHeat vs. ControlCu | 1.37540 | 0.13134 | 10.472 | **< 0.001** |
| CuControl vs. ControlHeat | -0.68336 | 0.13134 | -5.203 | **< 0.001** |
| CuCu vs. ControlHeat | -0.02513 | 0.13134 | -0.191 | 1.00000 |
| CuHeat vs. ControlHeat | -0.14173 | 0.09570 | -1.481 | 0.85025 |
| HeatControl vs. ControlHeat | 0.49523 | 0.13323 | 3.717 | **0.00586** |
| HeatCu vs. ControlHeat | 0.33371 | 0.13318 | 2.506 | 0.21194 |
| HeatHeat vs. ControlHeat | 1.41390 | 0.09570 | 14.774 | **< 0.001** |
| CuCu vs. CuControl | 0.65823 | 0.13134 | 5.012 | **< 0.001** |
| CuHeat vs. CuControl | 0.54163 | 0.13134 | 4.124 | **0.00114** |
| HeatControl vs. CuControl | 1.17859 | 0.09828 | 11.992 | **< 0.001** |
| HeatCu vs. CuControl | 1.01707 | 0.13318 | 7.637 | **< 0.001** |
| HeatHeat vs. CuControl | 2.09727 | 0.13134 | 15.968 | **< 0.001** |
| CuHeat vs. CuCu | -0.11660 | 0.13134 | -0.888 | 0.99255 |
| HeatControl vs. CuCu | 0.52037 | 0.13323 | 3.906 | **0.00278** |
| HeatCu vs. CuCu | 0.35885 | 0.09821 | 3.654 | **0.00714** |
| HeatHeat vs. CuCu | 1.43904 | 0.13134 | 10.957 | **< 0.001** |
| HeatControl vs. CuHeat | 0.63696 | 0.13323 | 4.781 | **< 0.001** |
| HeatCu vs. CuHeat | 0.47544 | 0.13318 | 3.570 | **0.00975** |
| HeatHeat vs. CuHeat | 1.55564 | 0.09570 | 16.255 | **< 0.001** |
| HeatCu vs. HeatControl | -0.16152 | 0.13504 | -1.196 | 0.95162 |
| HeatHeat vs. HeatControl | 0.91867 | 0.13323 | 6.895 | **< 0.001** |
| HeatHeat vs. HeatCu | 1.08019 | 0.13318 | 8.111 | **< 0.001** |

**Table S2.** General linear mixed effects model for population growth rate on the reduced dataset, omitting the clones of the control and Cu-selected populations that failed to survive the Cu + Heat treatment. Common garden treatments Cu, Heat and Selection history (SH) were specified as fixed factors, whereas Clone and Population ID as random factors. Bold P-values refer to significant effects (α = 0.05).

| **Dependent variable** | **Factor** | **SS** | **MS** | **NumDF** | **DenDF** | **F** | **P** |
| --- | --- | --- | --- | --- | --- | --- | --- |
| Population growth rate | Cu | 0.4 | 0.4 | 1 | 45.0 | 7 | **0.010** |
|  | Heat | 14.0 | 14.0 | 1 | 45.0 | 247 | **<0.001** |
|  | SH | 0.6 | 0.3 | 2 | 4.6 | 5 | 0.069 |
|  | Cu x Heat | 0.2 | 0.2 | 1 | 45.0 | 3 | 0.093 |
|  | Heat X SH | 2.4 | 1.2 | 2 | 45.0 | 21 | **<0.001** |
|  | Cu x SH | 0.2 | 0.1 | 2 | 45.0 | 2 | 0.139 |
|  | Cu x Heat x SH | 0.5 | 0.3 | 2 | 45.0 | 5 | **0.014** |

**Table S3.** Tukey post hoc pairwise comparisons for population growth rate among Cu x Heat × Selection history level combinations based on the reduced dataset, omitting the clones of the control and Cu-selected populations that failed to survive the Cu + Heat treatment. ‘CGT-SHC’ refers to specific common garden treatment – selection history combinations.

| **Multiple Comparisons**  **(CGT-SH vs CGT-SH)** | **Estimate Std.** | **Error** | **z value** | **Pr(>\|z\|)** |
| --- | --- | --- | --- | --- |
| ControlCu vs. ControlControl | 0.14589 | 0.215314 | 0.678 | 0.9999 |
| ControlHeat vs. ControlControl | 0.061959 | 0.215314 | 0.288 | 1 |
| Cu+HeatControl vs. ControlControl | 0.641378 | 0.140562 | 4.563 | **<0.01** |
| Cu+HeatCu vs. ControlControl | 0.569112 | 0.215314 | 2.643 | 0.2213 |
| Cu+HeatHeat vs. ControlControl | 1.255976 | 0.215314 | 5.833 | **<0.01** |
| CuControl vs. ControlControl | -0.606034 | 0.140562 | -4.312 | **<0.01** |
| CuCu vs. ControlControl | 0.149715 | 0.215314 | 0.695 | 0.9999 |
| CuHeat vs. ControlControl | -0.087465 | 0.215314 | -0.406 | 1 |
| HeatControl vs. ControlControl | 0.57246 | 0.140562 | 4.073 | **<0.01** |
| HeatCu vs. ControlControl | 0.565694 | 0.215314 | 2.627 | 0.2291 |
| HeatHeat vs. ControlControl | 1.555429 | 0.215314 | 7.224 | **<0.01** |
| ControlHeat vs. ControlCu | -0.083931 | 0.214099 | -0.392 | 1 |
| Cu+HeatControl vs. ControlCu | 0.495487 | 0.215314 | 2.301 | 0.4292 |
| Cu+HeatCu vs. ControlCu | 0.423222 | 0.140562 | 3.011 | 0.0881 |
| Cu+HeatHeat vs. ControlCu | 1.110086 | 0.214099 | 5.185 | **<0.01** |
| CuControl vs. ControlCu | -0.751924 | 0.215314 | -3.492 | **0.0204** |
| CuCu vs. ControlCu | 0.003824 | 0.140562 | 0.027 | 1 |
| CuHeat vs. ControlCu | -0.233355 | 0.214099 | -1.09 | 0.9934 |
| HeatControl vs. ControlCu | 0.42657 | 0.215314 | 1.981 | 0.663 |
| HeatCu vs. ControlCu | 0.419804 | 0.140562 | 2.987 | 0.0948 |
| HeatHeat vs. ControlCu | 1.409539 | 0.214099 | 6.584 | **<0.01** |
| Cu+HeatControl vs. ControlHeat | 0.579418 | 0.215314 | 2.691 | 0.1994 |
| Cu+HeatCu vs. ControlHeat | 0.507153 | 0.214099 | 2.369 | 0.3811 |
| Cu+HeatHeat vs. ControlHeat | 1.194016 | 0.140562 | 8.495 | **<0.01** |
| CuControl vs. ControlHeat | -0.667993 | 0.215314 | -3.102 | 0.0683 |
| CuCu vs. ControlHeat | 0.087755 | 0.214099 | 0.41 | 1 |
| CuHeat vs. ControlHeat | -0.149424 | 0.140562 | -1.063 | 0.9946 |
| HeatControl vs. ControlHeat | 0.510501 | 0.215314 | 2.371 | 0.38 |
| HeatCu vs. ControlHeat | 0.503735 | 0.214099 | 2.353 | 0.3924 |
| HeatHeat vs. ControlHeat | 1.493469 | 0.140562 | 10.625 | **<0.01** |
| Cu+HeatCu vs. Cu+HeatControl | -0.072265 | 0.215314 | -0.336 | 1 |
| Cu+HeatHeat vs. Cu+HeatControl | 0.614598 | 0.215314 | 2.854 | 0.1339 |
| CuControl vs. Cu+HeatControl | -1.247411 | 0.140562 | -8.874 | **<0.01** |
| CuCu vs. Cu+HeatControl | -0.491663 | 0.215314 | -2.283 | 0.4412 |
| CuHeat vs. Cu+HeatControl | -0.728842 | 0.215314 | -3.385 | **0.0282** |
| HeatControl vs. Cu+HeatControl | -0.068918 | 0.140562 | -0.49 | 1 |
| HeatCu vs. Cu+HeatControl | -0.075683 | 0.215314 | -0.352 | 1 |
| HeatHeat vs. Cu+HeatControl | 0.914051 | 0.215314 | 4.245 | **<0.01** |
| Cu+HeatHeat vs. Cu+HeatCu | 0.686863 | 0.214099 | 3.208 | 0.0503 |
| CuControl vs. Cu+HeatCu | -1.175146 | 0.215314 | -5.458 | **<0.01** |
| CuCu vs. Cu+HeatCu | -0.419398 | 0.140562 | -2.984 | 0.0953 |
| CuHeat vs. Cu+HeatCu | -0.656577 | 0.214099 | -3.067 | 0.0746 |
| HeatControl vs. Cu+HeatCu | 0.003347 | 0.215314 | 0.016 | 1 |
| HeatCu vs. Cu+HeatCu | -0.003418 | 0.140562 | -0.024 | 1 |
| HeatHeat vs. Cu+HeatCu | 0.986316 | 0.214099 | 4.607 | **<0.01** |
| CuControl vs. Cu+HeatHeat | -1.862009 | 0.215314 | -8.648 | **<0.01** |
| CuCu vs. Cu+HeatHeat | -1.106261 | 0.214099 | -5.167 | **<0.01** |
| CuHeat vs. Cu+HeatHeat | -1.343441 | 0.140562 | -9.558 | **<0.01** |
| HeatControl vs. Cu+HeatHeat | -0.683516 | 0.215314 | -3.175 | 0.0559 |
| HeatCu vs. Cu+HeatHeat | -0.690281 | 0.214099 | -3.224 | **0.0472** |
| HeatHeat vs. Cu+HeatHeat | 0.299453 | 0.140562 | 2.13 | 0.553 |
| CuCu vs. CuControl | 0.755748 | 0.215314 | 3.51 | **0.0184** |
| CuHeat vs. CuControl | 0.518569 | 0.215314 | 2.408 | 0.355 |
| HeatControl vs. CuControl | 1.178494 | 0.140562 | 8.384 | **<0.01** |
| HeatCu vs. CuControl | 1.171728 | 0.215314 | 5.442 | **<0.01** |
| HeatHeat vs. CuControl | 2.161462 | 0.215314 | 10.039 | **<0.01** |
| CuHeat vs. CuCu | -0.237179 | 0.214099 | -1.108 | 0.9924 |
| HeatControl vs. CuCu | 0.422745 | 0.215314 | 1.963 | 0.6756 |
| HeatCu vs. CuCu | 0.41598 | 0.140562 | 2.959 | 0.1013 |
| HeatHeat vs. CuCu | 1.405714 | 0.214099 | 6.566 | **<0.01** |
| HeatControl vs. CuHeat | 0.659925 | 0.215314 | 3.065 | 0.0768 |
| HeatCu vs. CuHeat | 0.653159 | 0.214099 | 3.051 | 0.0784 |
| HeatHeat vs. CuHeat | 1.642894 | 0.140562 | 11.688 | **<0.01** |
| HeatCu vs. HeatControl | -0.006766 | 0.215314 | -0.031 | 1 |
| HeatHeat vs. HeatControl | 0.982969 | 0.215314 | 4.565 | **<0.01** |
| HeatHeat vs. HeatCu | 0.989734 | 0.214099 | 4.623 | **<0.01** |

**Table S4.** Tukey post hoc pairwise comparisons for mortality among Treatment × Selection history level combinations. ‘CGT-SHC’ refers to specific common garden treatment – selection history combinations.

| **Multiple Comparisons**  **(CGT-SH vs CGT-SH)** | **Estimate** | **Std. Error** | **z value** | **Pr(>\|z\|)** |
| --- | --- | --- | --- | --- |
| ControlCu vs. ControlControl | 0.08553 | 0.30633 | 0.279 | 1.0000 |
| ControlHeat vs. ControlControl | -0.06502 | 0.31772 | -0.205 | 1.0000 |
| CuControl vs. ControlControl | 3.26288 | 0.20117 | 16.220 | **<0.001** |
| CuCu vs. ControlControl | 1.36103 | 0.27572 | 4.936 | **<0.001** |
| CuHeat vs. ControlControl | 1.79963 | 0.27126 | 6.634 | **<0.001** |
| HeatControl vs. ControlControl | 2.02004 | 0.19870 | 10.166 | **<0.001** |
| HeatCu vs. ControlControl | 2.29242 | 0.26565 | 8.629 | **<0.001** |
| HeatHeat vs. ControlControl | 0.39273 | 0.26932 | 1.458 | 0.8387 |
| ControlHeat vs. ControlCu | -0.15055 | 0.30078 | -0.501 | 0.9998 |
| CuControl vs. ControlCu | 3.17735 | 0.24689 | 12.869 | **<0.001** |
| CuCu vs. ControlCu | 1.27549 | 0.18602 | 6.857 | **<0.001** |
| CuHeat vs. ControlCu | 1.71409 | 0.25120 | 6.824 | **<0.001** |
| HeatControl vs. ControlCu | 1.93451 | 0.24590 | 7.867 | **<0.001** |
| HeatCu vs. ControlCu | 2.20689 | 0.17438 | 12.655 | **<0.001** |
| HeatHeat vs. ControlCu | 0.30720 | 0.24917 | 1.233 | 0.9311 |
| CuControl vs. ControlHeat | 3.32790 | 0.26083 | 12.759 | **<0.001** |
| CuCu vs. ControlHeat | 1.42604 | 0.26955 | 5.291 | **<0.001** |
| CuHeat vs. ControlHeat | 1.86464 | 0.19940 | 9.351 | **<0.001** |
| HeatControl vs. ControlHeat | 2.08505 | 0.25986 | 8.024 | **<0.001** |
| HeatCu vs. ControlHeat | 2.35743 | 0.25932 | 9.091 | **<0.001** |
| HeatHeat vs. ControlHeat | 0.45775 | 0.19714 | 2.322 | 0.2762 |
| CuCu vs. CuControl | -1.90186 | 0.20766 | -9.158 | **<0.001** |
| CuHeat vs. CuControl | -1.46326 | 0.20166 | -7.256 | **<0.001** |
| HeatControl vs. CuControl | -1.24284 | 0.08389 | -14.815 | **<0.001** |
| HeatCu vs. CuControl | -0.97046 | 0.19400 | -5.002 | **<0.001** |
| HeatHeat vs. CuControl | -2.87015 | 0.19907 | -14.418 | **<0.001** |
| CuHeat vs. CuCu | 0.43860 | 0.21281 | 2.061 | 0.4378 |
| HeatControl vs. CuCu | 0.65901 | 0.20647 | 3.192 | **0.0302** |
| HeatCu vs. CuCu | 0.93139 | 0.11155 | 8.349 | **<0.001** |
| HeatHeat vs. CuCu | -0.96829 | 0.21040 | -4.602 | **<0.001** |
| HeatControl vs. CuHeat | 0.22041 | 0.20041 | 1.100 | 0.9641 |
| HeatCu vs. CuHeat | 0.49279 | 0.19969 | 2.468 | 0.2042 |
| HeatHeat vs. CuHeat | -1.40689 | 0.10746 | -13.092 | **<0.001** |
| HeatCu vs. HeatControl | 0.27238 | 0.19263 | 1.414 | 0.8604 |
| HeatHeat vs. HeatControl | -1.62731 | 0.19776 | -8.229 | **<0.001** |
| HeatHeat vs. HeatCu | -1.89969 | 0.19708 | -9.639 | **<0.001** |

**Table S5.** Tukey post hoc pairwise comparisons for fecundity among Treatment × Selection history level combinations. ‘CGT-SHC’ refers to specific common garden treatment – selection history combinations.

| **Multiple Comparisons**  **(CGT-SH vs CGT-SH)** | **Estimate** | **Std. Error** | **z value** | **Pr(>\|z\|)** |
| --- | --- | --- | --- | --- |
| ControlCu vs. ControlControl | -0.05420 | 0.11075 | -0.489 | 0.99990 |
| ControlHeat vs. ControlControl | 0.06371 | 0.11075 | 0.575 | 0.99965 |
| CuControl vs. ControlControl | -0.69108 | 0.08006 | -8.632 | **< 0.001** |
| CuCu vs. ControlControl | -0.37983 | 0.11075 | -3.430 | **0.01551** |
| CuHeat vs. ControlControl | -0.49382 | 0.11208 | -4.406 | **< 0.001** |
| HeatControl vs. ControlControl | -0.47209 | 0.07778 | -6.070 | **< 0.001** |
| HeatCu vs. ControlControl | -0.45975 | 0.11363 | -4.046 | **0.00154** |
| HeatHeat vs. ControlControl | -0.29602 | 0.11075 | -2.673 | 0.14173 |
| ControlHeat vs. ControlCu | 0.11791 | 0.10928 | 1.079 | 0.97291 |
| CuControl vs. ControlCu | -0.63688 | 0.11250 | -5.661 | **< 0.001** |
| CuCu vs. ControlCu | -0.32564 | 0.07447 | -4.373 | **< 0.001** |
| CuHeat vs. ControlCu | -0.43962 | 0.11063 | -3.974 | **0.00207** |
| HeatControl vs. ControlCu | -0.41789 | 0.11075 | -3.773 | **0.00448** |
| HeatCu vs. ControlCu | -0.40556 | 0.07868 | -5.154 | **< 0.001** |
| HeatHeat vs. ControlCu | -0.24182 | 0.10928 | -2.213 | 0.36767 |
| CuControl vs. ControlHeat | -0.75479 | 0.11250 | -6.709 | **< 0.001** |
| CuCu vs. ControlHeat | -0.44354 | 0.10928 | -4.059 | **0.00159** |
| CuHeat vs. ControlHeat | -0.55753 | 0.07644 | -7.294 | **< 0.001** |
| HeatControl vs. ControlHeat | -0.53580 | 0.11075 | -4.838 | **< 0.001** |
| HeatCu vs. ControlHeat | -0.52346 | 0.11219 | -4.666 | **< 0.001** |
| HeatHeat vs. ControlHeat | -0.35973 | 0.07447 | -4.831 | **< 0.001** |
| CuCu vs. CuControl | 0.31125 | 0.11250 | 2.767 | 0.11221 |
| CuHeat vs. CuControl | 0.19726 | 0.11381 | 1.733 | 0.69839 |
| HeatControl vs. CuControl | 0.21899 | 0.08006 | 2.735 | 0.12167 |
| HeatCu vs. CuControl | 0.23133 | 0.11534 | 2.006 | 0.50753 |
| HeatHeat vs. CuControl | 0.39506 | 0.11250 | 3.512 | **0.01197** |
| CuHeat vs. CuCu | -0.11399 | 0.11063 | -1.030 | 0.97964 |
| HeatControl vs. CuCu | -0.09226 | 0.11075 | -0.833 | 0.99497 |
| HeatCu vs. CuCu | -0.07992 | 0.07868 | -1.016 | 0.98152 |
| HeatHeat vs. CuCu | 0.08381 | 0.10928 | 0.767 | 0.99717 |
| HeatControl vs. CuHeat | 0.02173 | 0.11208 | 0.194 | 1.00000 |
| HeatCu vs. CuHeat | 0.03407 | 0.11351 | 0.300 | 1.00000 |
| HeatHeat vs. CuHeat | 0.19780 | 0.07644 | 2.588 | 0.17273 |
| HeatCu vs. HeatControl | 0.01234 | 0.11363 | 0.109 | 1.00000 |
| HeatHeat vs. HeatControl | 0.17607 | 0.11075 | 1.590 | 0.78794 |
| HeatHeat vs. HeatCu | 0.16373 | 0.11219 | 1.459 | 0.85682 |


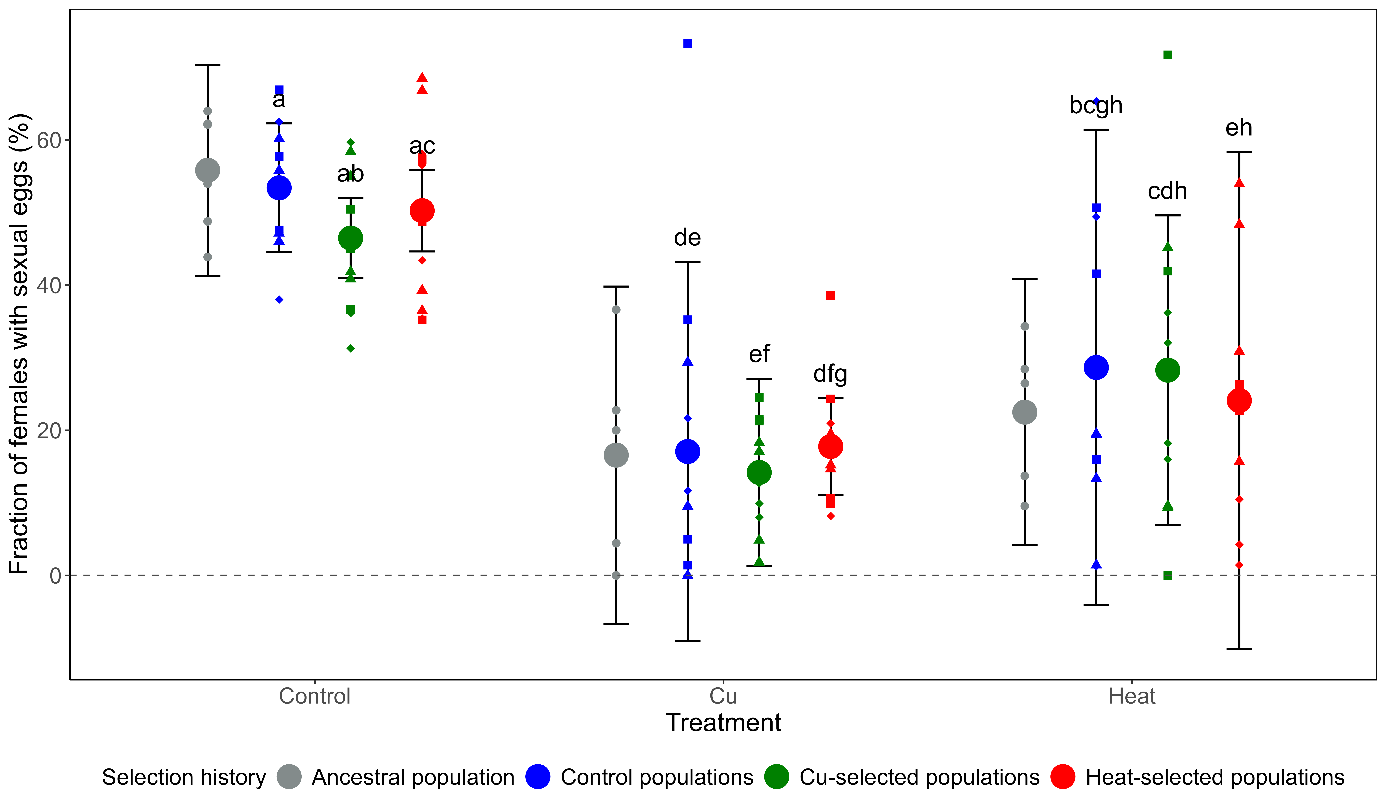


**Figure S2.** Fraction of females with sexual versus asexual eggs in Ancestral, Control, Cu- and Heat-selected populations in response to the Control, Cu, and Heat treatments of the common garden experiment. Symbols and error bars represent means and 95% confidence intervals across population replicates of the selection experiment. Letters denote differences according to post hoc Tuckey pairwise comparisons (alpha = 0.05). Note that, because ancestral populations did not align with the experimental design, they were not included in this posthoc comparison. Small symbols represent individual clones where different symbol types identify clones that originated from the same population in the selection experiment.

**Table S6.** Tukey post hoc pairwise comparisons for the counts of females with and without sexual eggs among Treatment × Selection history level combinations. ‘CGT-SHC’ refers to specific common garden treatment – selection history combinations.

| **Multiple Comparisons**  **(CGT-SH vs CGT-SH)** | **Estimate** | **Std. Error** | **z value** | **Pr(>\|z\|)** |
| --- | --- | --- | --- | --- |
| ControlCu vs. ControlControl | 0.43951 | 0.30924 | 1.421 | 0.83636 |
| ControlHeat vs. ControlControl | 0.26577 | 0.30940 | 0.859 | 0.99089 |
| CuControl vs. ControlControl | 1.89901 | 0.17739 | 10.705 | **< 0.001** |
| CuCu vs. ControlControl | 2.12977 | 0.32032 | 6.649 | **< 0.001** |
| CuHeat vs. ControlControl | 1.95792 | 0.32027 | 6.113 | **< 0.001** |
| HeatControl vs. ControlControl | 1.12696 | 0.09124 | 12.352 | **< 0.001** |
| HeatCu vs. ControlControl | 1.11609 | 0.31136 | 3.585 | **0.00610** |
| HeatHeat vs. ControlControl | 1.48814 | 0.30606 | 4.862 | **< 0.001** |
| ControlHeat vs. ControlCu | -0.17374 | 0.30636 | -0.567 | 0.99951 |
| CuControl vs. ControlCu | 1.45950 | 0.33946 | 4.299 | **< 0.001** |
| CuCu vs. ControlCu | 1.69026 | 0.12004 | 14.081 | **< 0.001** |
| CuHeat vs. ControlCu | 1.51841 | 0.31720 | 4.787 | **< 0.001** |
| HeatControl vs. ControlCu | 0.68745 | 0.30899 | 2.225 | 0.29613 |
| HeatCu vs. ControlCu | 0.67659 | 0.09389 | 7.206 | **< 0.001** |
| HeatHeat vs. ControlCu | 1.04863 | 0.30286 | 3.462 | **0.00969** |
| CuControl vs. ControlHeat | 1.63324 | 0.33963 | 4.809 | **< 0.001** |
| CuCu vs. ControlHeat | 1.86400 | 0.31739 | 5.873 | **< 0.001** |
| CuHeat vs. ControlHeat | 1.69215 | 0.12081 | 14.006 | **< 0.001** |
| HeatControl vs. ControlHeat | 0.86120 | 0.30916 | 2.786 | 0.07930 |
| HeatCu vs. ControlHeat | 0.85033 | 0.30834 | 2.758 | 0.08556 |
| HeatHeat vs. ControlHeat | 1.22237 | 0.07578 | 16.130 | **< 0.001** |
| CuCu vs. CuControl | 0.23076 | 0.34942 | 0.660 | 0.99852 |
| CuHeat vs. CuControl | 0.05891 | 0.34942 | 0.169 | 1.00000 |
| HeatControl vs. CuControl | -0.77204 | 0.17632 | -4.379 | **< 0.001** |
| HeatCu vs. CuControl | -0.78291 | 0.34121 | -2.294 | 0.25738 |
| HeatHeat vs. CuControl | -0.41087 | 0.33645 | -1.221 | 0.92351 |
| CuHeat vs. CuCu | -0.17185 | 0.32785 | -0.524 | 0.99973 |
| HeatControl vs. CuCu | -1.00280 | 0.32007 | -3.133 | **0.02885** |
| HeatCu vs. CuCu | -1.01367 | 0.12222 | -8.294 | **< 0.001** |
| HeatHeat vs. CuCu | -0.64163 | 0.31399 | -2.043 | 0.41047 |
| HeatControl vs. CuHeat | -0.83095 | 0.32002 | -2.597 | 0.12940 |
| HeatCu vs. CuHeat | -0.84182 | 0.31910 | -2.638 | 0.11685 |
| HeatHeat vs. CuHeat | -0.46978 | 0.11064 | -4.246 | **< 0.001** |
| HeatCu vs. HeatControl | -0.01087 | 0.31109 | -0.035 | 1.00000 |
| HeatHeat vs. HeatControl | 0.36117 | 0.30580 | 1.181 | 0.93622 |
| HeatHeat vs. HeatCu | 0.37204 | 0.30484 | 1.220 | 0.92376 |


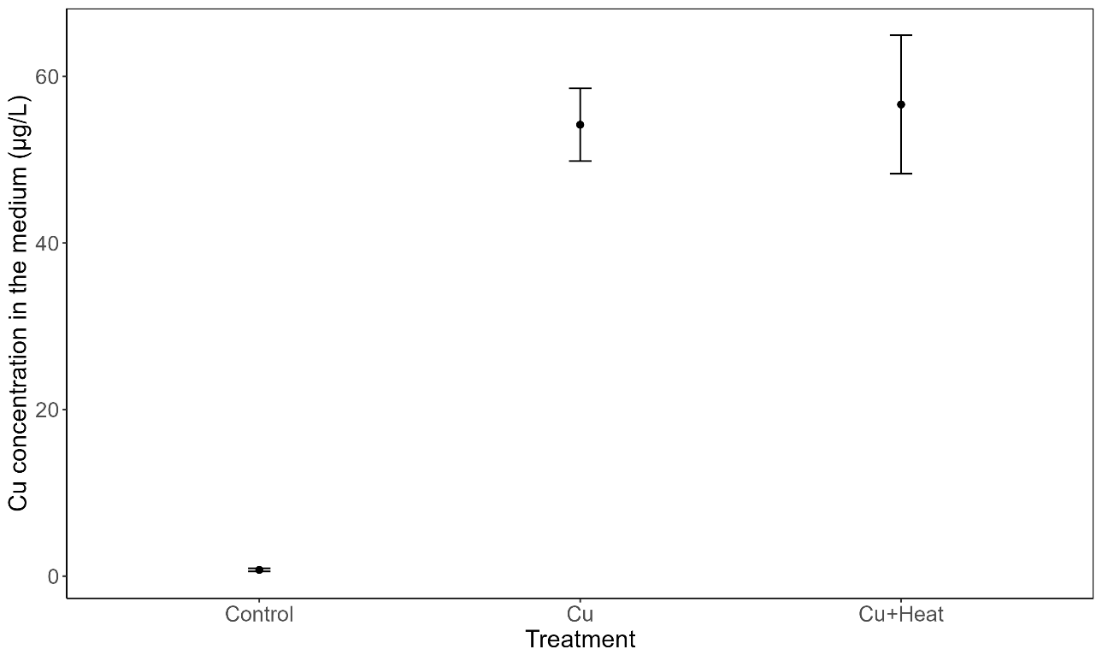


**Figure S3.** Cu concentration measured in the medium of rotifer populations subjected to Control, Cu and Cu + Heat treatments. Samples of the culture medium were daily collected during a period of five days. Error bars represent the 95% confidence interval of the mean.

**Reference**

Zhao, S. Y., Zhou, L., Chen, G., & Declerck, S. A. J. (2023). Rapidly evolving zooplankton in a salinizing world: To what extent does microevolutionary adaptation to one salt increase tolerance to another one? *Limnology and Oceanography*, *68*(50), 2576–2586. https://doi.org/10.1002/lno.12443
